# Hysteroscopic metroplasty for a complete uterine septum: step-by-step technique and surgical pearls

**DOI:** 10.1093/jscr/rjag527

**Published:** 2026-06-26

**Authors:** Ee Thong Lim, Mohammad Saaed ElFarran, Thomas Mosedale, Raj Mathur, Nikolaos Tsampras

**Affiliations:** Manchester University NHS Foundation Trust, Manchester, United Kingdom; Manchester University NHS Foundation Trust, Manchester, United Kingdom; Manchester University NHS Foundation Trust, Manchester, United Kingdom; Developmental Biology and Medicine, School of Medical Sciences, The University of Manchester, Manchester, United Kingdom; Developmental Biology and Medicine, School of Medical Sciences, The University of Manchester, Manchester, United Kingdom

**Keywords:** hysteroscopic metroplasty, hysteroscopic septum resection, septate uterus, uterine septum, uterine septum U2b, complete uterine septum

## Abstract

Septate uterus is the most common congenital uterine anomaly. It is associated with increased risks of adverse obstetric outcomes. Hysteroscopic metroplasty is the standard minimally invasive treatment in selected patients, although its effect on reproductive outcomes remains controversial. We present a case of hysteroscopic metroplasty in a 31-year-old woman with a complete uterine septum extending to the internal cervical os (class U2b^5^) and a history of preterm delivery at 26 weeks gestation. The procedure was performed using an operative hysteroscope with a bipolar Versapoint spring electrode. The septum was resected progressively under direct visualization until both ostia were identified at the same level and healthy pink myometrial tissue was seen. This video case report demonstrates a systematic approach to hysteroscopic metroplasty, providing practical guidance for surgeons performing this procedure. Careful patient selection and detailed counselling are essential to ensure that appropriate candidates understand the current evidence.

## Introduction

Septate uterus is the most common congenital uterine anomaly, accounting for ~35% of diagnosed uterine anomalies [1]. It results from incomplete resorption of the tissue between the Müllerian ducts during embryonic development [1, 2]. While the European Society of Human Reproduction and Embryology−European Society of Gynaecologic Endoscopy [3] classification defines a septate uterus as an internal indentation exceeding 50% of the uterine wall thickness—a criterion that may overestimate prevalence—the more recent Congenital Uterine Malformation by Experts consensus in 2018 proposes stricter criteria requiring an internal fundal indentation depth of at least 1 cm and an external indentation of less than 1 cm [1].

Septate uterus is associated with significantly increased risks of adverse obstetric outcomes, including spontaneous miscarriage, preterm delivery, foetal malpresentation, and decreased pregnancy rate [1, 2, 4, 5]. For selected patients, hysteroscopic metroplasty is the standard minimally invasive surgical treatment to improve reproductive outcome [1]. Despite these well-established associations, the role of hysteroscopic metroplasty in improving reproductive outcomes remains a subject of ongoing debate.

## Case presentation

We present a case of hysteroscopic metroplasty in a 31-year-old woman with a complete uterine septum extending to the internal cervical os (class U2b^5^) and a history of preterm delivery at 26 weeks gestation. A three-dimensional ultrasound scan was performed to confirm the findings. The primary goal of surgical intervention was to optimize conditions for a subsequent pregnancy to reach term, with evidence for preterm birth reduction following septum resection providing the rationale for correction. This video case report demonstrates a systematic approach to hysteroscopic metroplasty, highlighting key surgical steps and practical tips for safe and effective resection (Video 1).

## Surgical technique

Using an operative hysteroscope with a five-French working channel, saline hysteroscopy with hydrodilatation was performed to minimize cervical trauma and optimize visualization. Initial inspection revealed a midline septum arising from the uterine fundus as an avascular fibrous band dividing the uterine cavity. Under direct vision, the septum was divided using a bipolar Versapoint spring electrode. A prospective randomized trial has demonstrated that small-diameter hysteroscopy with the bipolar Versapoint electrode is as effective as unipolar resectoscope in terms of reproductive outcomes, while being associated with significantly shorter operating time, lower fluid absorption, and a reduced complication rate [6]. Resection was performed progressively from the distal portion of the septum toward the fundus, with the depth of division guided by the appearance of the underlying myometrium. Incision was continued until healthy pink myometrial tissue was visualized, indicating that the vascularized fundal muscle had been reached. Adequate resection was confirmed once both tubal ostia were visualized at the same level, restoring a single triangular uterine cavity. Care was taken throughout procedure to avoid uterine perforation and excessive thinning of the fundal myometrium. Hyaluronic acid gel barrier was applied at the end of procedure to reduce the risk of de novo intrauterine adhesions [7].

The procedure was uneventful, and the patient was discharged on the same day. Follow-up three-dimensional ultrasound confirmed successful resection of the septum with restoration of a normal uterine cavity.

## Discussion

The efficacy of hysteroscopic metroplasty in improving reproductive outcomes has been evaluated in several meta-analyses. Two large meta-analyses (*n* = 1589 and *n* = 659) have demonstrated significant reductions in miscarriage risk and foetal malpresentation, with preterm birth reduction specifically noted in women with partial septum [1, 3]. In contrast, a more recent meta-analysis by Liu *et al.* (*n* = 468) in 2024, which included only two randomized controlled trials and one cohort study, found no significant benefit for any reproductive outcome [2]. However, this study is limited by its smaller sample size and has been criticized for methodological limitations, highlighting the need for further high-quality research. None of the meta-analyses have demonstrated significant improvements in live birth or clinical pregnancy rates.

Given the limited and inconclusive evidence and the inherent risks associated with surgical intervention, hysteroscopic metroplasty should only be performed when clinical indications are present [8]. Long uterine septum can substantially reduce the effective volume of the uterine cavity; therefore, surgical correction may be justified in women with a history of late miscarriage or preterm birth. Recurrent miscarriages and primary infertility may be considered relative indications for intervention. In contrast, a previous term live birth is generally regarded as a relative contraindication to surgical treatment [9].

In conclusion, this video case report demonstrates a systematic approach to hysteroscopic metroplasty for complete uterine septum using bipolar electrode. The stepwise technique illustrated provides practical guidance for surgeons performing this procedure. Careful patient selection and detailed counselling are essential to ensure that appropriate candidates understand the current evidence, including the demonstrated benefits for miscarriage and malpresentation reduction, the absence of proven improvement in live birth rates, and the ongoing debate surrounding this intervention.

## Supplementary Material

Hysteroscopic_metroplasty_final_draft_(revised_submission)_rjag527
